# Longitudinal relationship between body fat percentage and risk of type 2 diabetes in Chinese adults: Evidence from the China Health and Nutrition Survey

**DOI:** 10.3389/fpubh.2022.1032130

**Published:** 2022-11-29

**Authors:** Siting Zhang, Hongru Jiang, Liusen Wang, Xiaofang Jia, Jiguo Zhang, Huijun Wang, Bing Zhang, Zhihong Wang, Gangqiang Ding

**Affiliations:** ^1^National Institute for Nutrition and Health, Chinese Center for Disease Control and Prevention, Beijing, China; ^2^Key Laboratory of Trace Elements and Nutrition, National Health Commission, Beijing, China

**Keywords:** body fat percentage, cut-off points, type 2 diabetes, obesity, China

## Abstract

**Objective:**

Body fat percentage (BF%) might be an alternative index of obesity which is the major risk factor for developing type 2 diabetes (T2D). We aim to longitudinally evaluated the relationship between BF% and risk of T2D.

**Methods:**

A sample of 5,595 adults aged 18–65 who participated in two waves of China Health and Nutrition Survey (CHNS 2015 and 2018) was analyzed. Two level mixed-effects modified Poisson regression with robust estimation of variance stratified by sex was used to evaluate the risk ratios (RRs) for T2D according to quintiles of BF%, and the curves of receiver operating characteristic (ROC) were plotted to identify the optimal total and trunk BF% cut-off points for predicting an increased T2D risk.

**Results:**

In males, compared with subjects in the first quintile of total BF%, those in the third (RR = 2.03, 95% CI 1.09–3.79), fourth (RR = 2.56, 95%CI 1.46–4.48), and fifth (RR = 2.16, 95%CI 1.22–3.82) quintile had higher risk of T2D after adjusting for all potential confounders (*p*-trend < 0.001). For females, the RR (95% CI) was 1.92 (1.14, 3.24) in the fifth quintile (*p*-trend = 0.014). Males and females with a trunk BF% >25.5 and 34.4% (≥ quintile 4), respectively, were at significantly increased risk of T2D (*p*-trend = 0.001). Besides, the optimal cut-off values of total and trunk BF% were 21.9 and 25.2% for males, and 36.7 and 30.3% for females, respectively.

**Conclusions:**

The incident risk of T2D significantly increased over specific level of total and trunk BF% in both Chinese males and females, and the optimal BF% cut-off values were valuable for clinical application of BF% based on sex difference, which may be a cost-effective implementation for prevention and treatment of T2D in China.

## Introduction

Urbanization, energy-dense diets, and physical inactivity, with a consequent epidemic of obesity have resulted in the rapid escalation of type 2 diabetes (T2D) around the world (1), particularly in some developing countries (2). Substantial evidence has demonstrated the cause effect of obesity on risk of T2D and insulin resistance (3). Due to their simplicity and ease, body mass index (BMI) and waist circumference (WC) have been routinely employed to identify obese individuals in most epidemiologic studies (4–6). However, these anthropometric measures are unable to directly evaluate body fat and its distribution, and likely to underestimate the prevalence of obesity among people with normal weight but high body fat (7). Body fat percentage (BF%) determined by impedance is increasingly advocated as a favorable measurement of body composition at home and in medical check-ups owing to its safety, simplicity and affordability.

A growing body of studies have shown significant associations between BF% and cardiovascular disease risk factors, such as diabetes mellitus, hypertension, and dyslipidemia, irrespective of BMI and abdominal obesity (8–10). For example, subjects with normal or obese BMI but excess BF% had increased risks of developing T2D compared to those with normal BF% in White populations (8), as well as in Chinese populations (10). Besides, one study with cross-sectional design indicated that obesity measured by BF% could be a better predictor of T2D risk than BMI (11). Although several studies have explored the threshold values of BF% for obesity in different ethnic groups, such as the American Association of Clinical Endocrinology (males: 25%, females: 35%) (12) and Korean adults (males: 21%, females: 37%) (13), the cut-off points of BF% that reflect an increased risk of obesity-related disease remains unclear. Thus, it is crucial to determine the optimal BF% values that indicated the increased risk of T2D.

Given the cross-section design of previous studies (13–15) and ethnicity difference in body fat distribution (16), the current evidence seems insufficient to propose appropriate modification of body fat mass and its distribution based on risk of T2D for Chinese population. Therefore, the present study aimed to examine the longitudinal associations of total and trunk BF% with risk of T2D, using data from the China Health and Nutrition Survey (CHNS), and further identify the optimal cut-off values of total and trunk BF% to predict an increase in T2D risk. Such findings would be valuable for developing and implementing public health actions to provide guidance for reduction and intervention of body fat and improve T2D status in Chinese individuals.

## Methods

### Study population

The current study utilized data from the CHNS, a population-based longitudinal survey with a focus on the relationships between sociological, economic and demographic changes and the effects on numerous nutritional and health status of social Chinese population. The CHNS was initiated in 1989 and carried out ten consecutive rounds of follow-up surveys during the period of 1991–2018, the details of the survey have been described elsewhere (17, 18).

**Figure 1:**
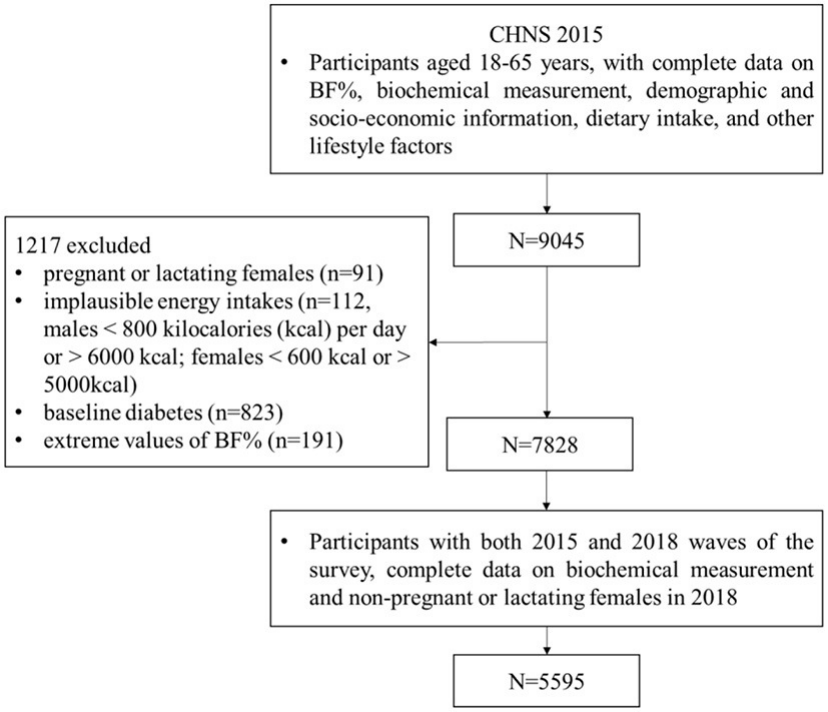
Flow chart of study population selection.

This study was based on the data from the 2015 and 2018 waves of the CHNS. We chose the adults aged 18 to 65 who participated in both rounds of survey and were not diabetes at baseline with complete data on BF%, biochemical measurements, demographic information, socio-economic information, dietary intake, and other lifestyle factors, and then we excluded pregnant or lactating females, those having implausible energy intakes, and those with extreme values of BF%. Figure 1 presents the flow chart of participant selection. We investigated the association between BF% at baseline and risk of T2D in 2018 considering the prospective nature with clear temporal characteristic. The final analysis therefore consisted of 5,595 participants (2,471 males; 3,124 females) clustered in 338 communities. Incidence of T2D means number of diabetes in 2018 among 5,595 adults without diabetes at baseline.

The survey protocol was approved by the Institutional Review Committees of the University of North Carolina at Chapel Hill and the Chinese Center for Disease Control and Prevention (No. 201524), and all subjects provided written informed consent.

### Assessment of body fat percentage

Trained health workers measured BF% including total BF%, trunk BF%, and arm and leg BF%, using a body composition analyzer (TANITA BC601) with the participants in lightweight clothing and without shoes, in the fasting state and before working in the morning. Based on the bioelectrical impedance analysis (BIA), BF% was calculated to the nearest 0.1% according to a proprietary algorithm that required age, sex, height, and physical activity level inputs by investigators. The method has been validated previously and employed regularly in other studies (17, 19). The current study recorded the total BF% (ratio of total body fat mass and total body mass) and trunk BF% (ratio of trunk fat mass and trunk mass) separately, and performed quintiles of them for analysis.

### Diagnosis of type 2 diabetes

Experienced nurses or phlebotomists collected overnight fasting blood samples *via* venipuncture. Blood samples were centrifuged within 3 h and preserved at −2~8°C refrigerator for short-term storage in order to obtain reliable test results for later laboratory analysis. Fasting blood glucose (FPG) and glycated hemoglobin A1c (HbA_1c_) were detected by GOD-PAP (Randox, UK) and HLC/HLC/HPLC (Tosoh, Japan/ Bio-Rad, USA/ Primus, USA) methods, respectively.

Based on the guideline for the prevention and treatment of type 2 diabetes mellitus in China (2020 edition) (20) and the World Health Organization criteria for diabetes mellitus (21), the diagnostic criterion for T2D was fasting glucose ≥7.0 mmol/L or HbA1c ≥6.5%, or a self-reported diagnosis of diabetes and treatment with antidiabetic pharmacotherapy.

### Assessment of covariates

Information on socio-demographic and lifestyle variables were collected through standard questionnaires by trained interviewers, and only baseline covariates in round 2015 were assessed in the present study. The following variables were included: age (in years); per capita family income (tertiles: low, medium, high); individual educational level (primary school and below, completed middle school, high school and above); residence (rural and urban); smoking and alcohol drinking status (current vs. former or non-); community urbanization index (score) calculated based on 12 dimensions of the community level including physical, social, economic, cultural and sanitary environments (22); sleep duration (6–9 h vs. <6 h or 9 h) (23); physical activity (in MET-h/week) referred to the Compendium of Physical Activities (24). In addition, the intake of other dietary factors were also regarded as potential confounders, including total energy intake (TEI), percentage of total energy comes from fat, dietary fiber (25), calcium (26), magnesium (27), and Vitamin C, which were assessed by three consecutive days of 24-h recalls for each individual and the weighing of seasonings in the household inventory over the same period.

### Statistical analysis

First, as shown in Supplementary Table 1, we performed statistical interaction tests between BF% and sex, and found significant interaction. We categorized the total and trunk BF% into five levels (quintiles of BF%) by sex, respectively. Baseline characteristics of participants were summarized and examined by Chi-square test for categorical variables, and Wilcoxon rank-sum and Kruskal-Wallis H test for non-normally distributed continuous variables.

To evaluate the association between the quintiles of total and trunk BF% and risk of T2D, a modified Poisson regression with robust (sandwich) estimation of variance was performed, which is an appropriate and reliable approach to estimate relative risk for the binary outcomes (28). Also, considering the hierarchical data structure of the CHNS, we used a two-level mixed-effects modified Poisson regression with robust (sandwich) estimation of variance to estimate the risk ratio (RRs) of T2D, taking communities as the second level and individual as the first level. We constructed three sequential models for analysis: Model 1 adjusted for no covariates; Model 2 adjusted for age, income level, education, residence, urbanicity index, physical activity, sleep duration, smoking and alcohol drinking; Model 3 further adjusted for TEI, percentage of total energy comes from fat, fiber, and other related dietary factors. In addition, linear trends across increasing categories of total and trunk BF% were assessed by assigning median values to levels of total and trunk BF%, and the variable modeled as a continuous term.

Furthermore, we plotted the curves of receiver operating characteristic (ROC) for the total and trunk BF% by sex to identify the cut-off points of BF% that predicted risk of T2D. Sensitivity, also known as true positive rate, reflects the ability of a screening test to detect patients; specificity, also known as true negative rate, reflects the ability of a screening test to identify non-patients. An ROC curve is produced by plotting a list of sensitivity on the y-axis against “1 – specificity” on the x-axis for different values of a continuous test measure. Two methods were used to determine the optimal cut-off points, which were the Youden's index reaching its maximum value (sensitivity + specificity – 1) and the shortest distance from the corner. The area under the curve (AUC) shows the authenticity of a test to classify the participants as likely to have disease or not, and the value of AUC is usually used to compare overall performances of different screening test, which is between 0 and 1, the closer it is to 1, the higher its application value (29). Additionally, potential covariates including socio-demographic, lifestyle and dietary variables were also adjusted among the ROC curves.

All statistical analyses were conducted using SAS version 9.4 (SAS Institute Inc., Cary, NC, USA) and Stata version 15SE (Stata Corp., College Station, TX, USA). Two-tailed *p* < 0.05 was considered statistically significant.

## Results

### Baseline characteristics

The baseline characteristics of participants across total BF% levels by sex are summarized in Tables 1A,B, respectively. Females tended to have an obviously higher total BF% than males. The mean age of the participants were 46.3 (10.4) years. Both males and females with higher total BF% levels were older. It is notable that males whose total BF% were higher tended to have lower physical activity levels, higher income levels and educational levels, and live in urban area (*p* < 0.05). On the contrary, females with higher total BF% had lower socioeconomic status (*p* < 0.05).

**Table 1A:** Baseline characteristics of adult males according to the quintiles of the total BF%, CHNS (*n* = 2,471).

**Baseline characteristics**	**Total**	**Q1**	**Q2**	**Q3**	**Q4**	**Q5**	***p*-value**
		**< 17.2%**	**17.2–21.1%**	**21.1–24.0%**	**24.0–27.3%**	**≥27.3%**	
Age (years)	46.3 ± 10.4	44.2 ± 11.5	47.2 ± 10.4	46.5 ± 10.0	46.1 ± 10.0	47.3 ± 9.8	<0.001
Income level (%)							<0.001
Low	31.8	39.4	34.7	27.2	28.4	29.4	
Medium	33.5	32.7	31.5	34.4	34.9	34.0	
High	34.7	27.9	33.8	38.4	36.7	36.6	
Education level (%)							<0.001
Primary school and below	16.5	21.9	17.6	16.1	13.7	13.3	
Middle school	39.1	46.3	38.7	36.8	35.9	37.6	
High school and above	44.4	31.8	43.7	47.1	50.4	49.1	
Residence (%)							<0.001
Rural	66.4	79.7	71.5	63.4	60.1	57.3	
Urban	33.6	20.3	28.5	36.6	39.9	42.7	
Urbanicity index	71.4 ± 17.6	66.0 ± 16.9	70.0 ± 17.2	73.0 ± 17.6	74.0 ± 17.6	74.2 ± 17.5	<0.001
Smoking (%)							0.061
Former/non-smoker	42.9	38.7	40.7	44.7	43.2	47.3	
Current smoker	57.1	61.3	59.3	55.3	56.9	52.7	
Alcohol drinking (%)							0.632
Former/non-drinker	40.2	42.4	37.9	40.3	41.1	39.0	
Current drinker	59.8	57.6	62.1	59.7	58.9	61.0	
Sleep duration (%)							0.647
6~9 h	83.9	85.4	82.4	85.2	83.5	83.3	
< 6/>9 h	16.1	14.6	17.6	14.8	16.5	16.7	
Physical activity (MET h/week)	184.4 ± 175.6	213.2 ± 203.8	188.8 ± 186.3	189.6 ± 179.5	166.3 ± 156.1	164.5 ± 141.6	<0.001
Dietary intake							
TEI (kcal/d)	2,189.1 ± 788.2	2,206.6 ± 833.3	2,161.8 ± 805.4	2,178.2 ± 801.5	2,166.7 ± 769.8	2,232.4 ± 728.6	0.146
Fat (% of total energy)	34.4 ± 12.0	34.5 ± 12.5	34.3 ± 12.3	34.5 ± 11.9	34.5 ± 12.0	34.1 ± 11.5	0.949
Fiber (g/d)	12.8 ± 8.8	13.0 ± 9.9	12.3 ± 8.1	12.7 ± 7.9	12.3 ± 7.1	13.5 ± 10.3	0.297
Calcium (mg/d)	372.1 ± 202.7	373.0 ± 202.3	356.6 ± 183.6	359.4 ± 190.2	380.0 ± 218.4	391.3 ± 215.5	0.043
Magnesium (mg/d)	288.3 ± 131.4	287.8 ± 125.5	285.2 ± 147.7	282.0 ± 115.3	284.7 ± 129.5	301.8 ± 135.9	0.039
Vitamin C (mg/d)	75.4 ± 111.1	72.3 ± 73.1	75.8 ± 123.1	74.8 ± 109.5	76.4 ± 102.1	77.8 ± 137.0	0.749

**Table 1B:** Baseline characteristics of adult females according to the quintiles of the total BF%, CHNS (*n* = 3,124).

**Baseline characteristics**	**Total**	**Q1**	**Q2**	**Q3**	**Q4**	**Q5**	***p*-value**
		**< 27.6%**	**27.6–31.6%**	**31.6–34.9%**	**34.9–38.6%**	**≥38.6%**	
Age (years)	46.3 ± 10.2	41.9 ± 11.4	45.2 ± 10.2	47.0 ± 9.6	47.7 ± 9.3	49.6 ± 8.4	<0.001
Income level (%)							0.005
Low	34.6	33.0	32.1	35.3	35.0	37.3	
Medium	33.2	30.9	32.9	32.3	32.5	37.6	
High	32.2	36.1	35.0	32.4	32.5	25.1	
Education level (%)							<0.001
Primary school and below	28.2	23.8	25.5	26.2	30.0	35.3	
Middle school	36.1	31.6	35.0	38.3	37.1	38.6	
High school and above	35.7	44.6	39.5	35.5	32.9	26.1	
Residence (%)							0.185
Rural	65.2	62.0	63.9	65.3	67.2	67.7	
Urban	34.8	38.0	36.1	34.7	32.8	32.3	
Urbanicity index	72.0 ± 17.2	72.0 ± 17.5	72.7 ± 17.2	72.1 ± 16.8	72.2 ± 17.3	70.8 ± 17.3	0.376
Smoking (%)							0.018
Former/non-smoker	98.5	97.2	99.4	98.2	98.4	99.2	
Current smoker	1.5	2.8	0.6	1.8	1.6	0.8	
Alcohol drinking (%)							0.521
Former/non-drinker	92.4	92.4	92.3	92.5	91.2	93.9	
Current drinker	7.6	7.6	7.7	7.5	8.8	6.1	
Sleep duration (%)							0.397
6~9 h	84.4	84.8	82.8	83.4	84.6	86.6	
< 6/>9 h	15.6	15.2	17.2	16.6	15.4	13.4	
Physical activity (MET h/week)	173.7 ± 160.3	171.9 ± 157.1	182.4 ± 164.9	171.5 ± 155.5	177.0 ± 169.3	165.7 ± 153.9	0.328
Dietary intake							
TEI (kcal/d)	1,824.8 ± 639.9	1,821.9 ± 644.5	1,803.1 ± 596.5	1,835.8 ± 657.4	1,837.0 ± 667.9	1,825.6 ± 631.4	0.978
Fat (% of total energy)	34.5 ± 12.3	33.7 ± 12.2	34.7 ± 12.0	34.9 ± 12.1	34.8 ± 12.8	34.5 ± 12.2	0.228
Fiber (g/d)	11.6 ± 7.6	11.6 ± 7.3	11.7 ± 8.7	11.6 ± 7.5	11.6 ± 7.3	11.6 ± 6.9	0.921
Calcium (mg/d)	334.8 ± 185.7	325.0 ± 187.9	339.2 ± 184.5	336.4 ± 171.6	331.3 ± 187.1	341.9 ± 196.7	0.166
Magnesium (mg/d)	250.5 ± 113.6	245.0 ± 100.0	247.1 ± 105.7	249.1 ± 120.5	253.3 ± 111.8	257.9 ± 127.8	0.565
Vitamin C (mg/d)	76.5 ± 154.6	70.3 ± 119.2	72.0 ± 151.4	87.3 ± 234.3	68.9 ± 59.2	84.2 ± 155.5	0.070

### Associations of total and trunk BF% with type 2 diabetes

A total of 282 participants were found to have T2D, and Table 2 presents the numbers of outcome events for each subtype stratified by sex. The associations between quintiles of total and trunk BF% and risk of T2D in Chinese adults stratified by sex are also shown in Table 2.

**Table 2:** Risk ratio (95% CI) of type 2 diabetes across the quintiles of the total and trunk BF% among Chinese adults aged 18–65, CHNS^a^.

		**Number of case/subjects**	**Model 1**	**Model 2**	**Model 3**
**Male**					
Total BF%					
Q1	< 17.2%	15/493	1	1	1
Q2	17.2–21.1%	15/499	0.97 (0.47, 2.01)	0.91 (0.44, 1.89)	0.92 (0.44, 1.94)
Q3	21.1–24.0%	31/486	2.10 (1.11, 3.96)^b^	2.00 (1.08, 3.73)^b^	2.03 (1.09, 3.79)^b^
Q4	24.0–27.3%	41/496	2.71 (1.52, 4.82)^c^	2.60 (1.48, 4.55)^c^	2.56 (1.46, 4.48)^c^
Q5	≥27.3%	35/497	2.31 (1.29, 4.11)^c^	2.17 (1.23, 3.84)^c^	2.16 (1.22, 3.82)^c^
*p* trend			<0.001	<0.001	<0.001
Trunk BF%					
Q1	< 17.2%	16/494	1	1	1
Q2	17.2–21.9%	21/501	1.26 (0.67, 2.38)	0.17 (0.62, 2.21)	1.18 (0.62, 2.22)
Q3	21.9–25.5%	21/481	1.32 (0.69, 2.53)	1.24 (0.66, 2.33)	1.25 (0.66, 2.34)
Q4	25.5–29.4%	43/500	2.66 (1.49, 4.75)^c^	2.51 (1.43, 4.41)^c^	2.46 (1.40, 4.31)^c^
Q5	≥29.4%	36/495	2.23 (1.30, 3.81)^c^	2.03 (1.19, 3.49)^b^	2.02 (1.18, 3.45)^b^
*p* trend			<0.001	<0.001	0.001
**Female**					
Total BF%					
Q1	< 27.6%	16/618	1	1	1
Q2	27.6–31.6%	29/623	1.81 (1.00, 3.28)	1.59 (0.88, 2.87)	1.55 (0.86, 2.77)
Q3	31.6–34.9%	22/626	1.36 (0.71, 2.58)	1.08 (0.57, 2.03)	1.07 (0.57, 2.01)
Q4	34.9–38.6%	33/637	1.99 (1.08, 3.63)^b^	1.56 (0.85, 2.84)	1.56 (0.86, 2.85)
Q5	≥38.6%	45/620	2.81 (1.66, 4.77)^c^	1.97 (1.16, 3.35)^b^	1.92 (1.14, 3.24)^b^
*p* trend			<0.001	0.012	0.014
Trunk BF%					
Q1	< 25.4%	13/625	1	1	1
Q2	25.4–30.5%	27/620	2.12 (1.12, 4.01)^b^	1.90 (1.01, 3.60)^b^	1.83 (0.98, 3.44)
Q3	30.5–34.4%	23/634	1.73 (0.89, 3.35)	1.48 (0.77, 2.83)	1.43 (0.75, 2.74)
Q4	34.4–39.0%	33/625	2.54 (1.34, 4.78)^c^	2.01 (1.07, 3.77)^b^	1.99 (1.06, 3.73)^b^
Q5	≥39.0%	49/620	3.81 (2.13, 6.82)^c^	2.71 (1.51, 4.86)^c^	2.58 (1.45, 4.60)^c^
*p* trend			<0.001	0.001	0.001

a A two-level mixed-effects Poisson regression with robust (sandwich) estimation of variance, taking community as the second level, and individual as the first level. Model 1 adjusted for no covariates. Model 2 adjusted for age, income level (categorical), education level (categorical), urbanized index, residence (categorical), smoking (categorical), alcohol drinking (categorical), physical activity and sleep duration (categorical). Model 3 additionally adjusted for TEI, percentage of total energy comes from total energy, dietary fiber, calcium, magnesium, and vitamin C intake.

b *p* < 0.05,

c *p* < 0.01. *p* trend was examined by assigning the median value of each quantile as a continuous variable.

After adjusting for all potential confounders, males in the third (21.1–24.0%), fourth (24.0–27.3%), and fifth (≥ 27.3%) quintiles of total BF% showed 2.03 (95% CI 1.09–3.79), 2.56 (95%CI 1.46–4.48), and 2.16 (95%CI 1.22–3.82) times the risk of T2D as compared with those in the lowest quintile (< 17.2%, *p*-trend < 0.001). In females, the RR (95% CI) for risk of T2D was1.92 (1.14, 3.24), when comparing the highest (≥ 38.6%) with the lowest (<27.6%) quintile (*p*-trend = 0.014). For trunk BF% of males, RRs (95%CI) of T2D were 2.46 (1.40, 4.31) and 2.02 (1.18, 3.45) in the fourth (25.5–29.4%) and fifth (≥ 29.4%) quintiles, respectively, as compared with the lowest quintile (<17.2%, *p*-trend = 0.001). For females, as compared the lowest quintile of trunk BF% (<25.4%), the RRs (95%CI) were 1.99 (1.06, 3.73) and 2.58 (1.45, 4.60) for risk of T2D in the fourth and fifth quintiles, respectively (*p*-trend = 0.001).

### Cut-off points of total and trunk BF% for risk of type 2 diabetes

**Figure 2:**
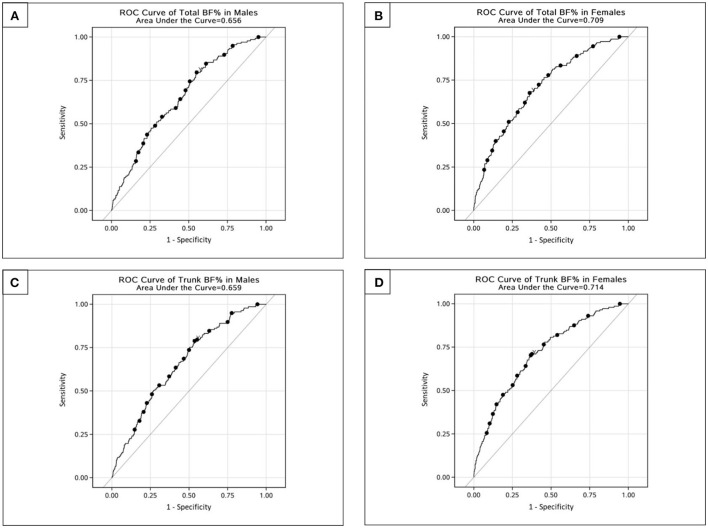
ROC curves for total and trunk BF% related to risk of type 2 diabetes among Chinese adults. **(A)** ROC curve of total BF% in males. **(B)** ROC curve of total BF% in females. **(C)** ROC curve of trunk BF% in males. **(D)** ROC curve of trunk BF% in females.

The ROC curves and the AUCs of the total and trunk BF% in relation to the risk of T2D were plotted and calculated to identity the values of total and trunk BF% that best predicted T2D risk (Figure 2). After adjusting for potential confounders, the AUCs for total BF% were 0.656 and 0.709, and the AUCs for trunk BF% were 0.659 and 0.714, respectively, in males and females.

The Youden's indices indicated that the optimal cut-off values of total and trunk BF% were 21.9% (sensitivity: 0.796; specificity: 0.449) and 25.2% (sensitivity: 0.788; specificity: 0.464) for males, and 36.7% (sensitivity: 0.676; specificity: 0.638) and 30.3% (sensitivity: 0.703; specificity: 0.632) for females, respectively. The shortest distance from the corner showed 13.2% (sensitivity: 0.555; specificity: 0.656) and 29.5% (sensitivity: 0.642; specificity: 0.583) for males, and 36.7% (sensitivity: 0.676; specificity: 0.638) and 23.4% (sensitivity: 0.697; specificity: 0.637) for females, respectively (Table 3).

**Table 3:** The appropriate cut-off points of total and trunk BF% for risk of type 2 diabetes among Chinese adults aged 18–65, CHNS.

	**Cut-off (%)**	**Sensitivity**	**Specificity**
Male	Youden's index		
Total BF%	21.9	0.796	0.449
Trunk BF%	25.2	0.788	0.464
Female			
Total BF%	36.7	0.676	0.638
Trunk BF%	30.3	0.703	0.632
Male	The shortest distance from the corner		
Total BF%	13.2	0.555	0.656
Trunk BF%	29.5	0.642	0.583
Female			
Total BF%	36.7	0.676	0.638
Trunk BF%	23.4	0.697	0.637

## Discussion

In this longitudinal prospective cohort study, we observed that males with total BF% more than 21.1% (≥ quintile 3) and trunk BF% more than 25.5% (≥ quintile 4), and females with total BF% more than 38.6% (quintile 5) and trunk BF% more than 34.4% (≥ quintile 4) had the significantly increased risk for T2D as compared with the subjects in quintile 1 group. Moreover, the optimal cut-off points determined by Youden's index and the shortest distance from the corner were different. For BF%, increased sensitivity for risk of T2D may promote physical activity and healthy lifestyle, whereas relatively wide margin for false positives may not lead to inappropriate treatment or serious physical, mental and financial burden. Therefore, it may be appropriate to choose the Youden's index to determine the optimal cut-off points which tend to comprehensively reflect the total ability of a screening test to detect patients or non-patients. The cut-off values of total and trunk BF% were 21.9% (sensitivity: 0.796; specificity: 0.449) and 25.2% (sensitivity: 0.788; specificity: 0.464) for males and 36.7% (sensitivity: 0.676; specificity: 0.638) and 30.3% (sensitivity: 0.703; specificity: 0.632) for females, respectively, based on the Youden's index.

Epidemiological evidence supported that body fat was significantly associated with the risk of obesity-related T2D, but the cut-off values varied in the different populations. Macek et al. (14) cross-sectionally identified the optimal BF% cut-off points for diabetes were 25.5% for males and 40.0% for females based on a sample of 4,735 Polish adults aged 45–64 years old. Zhu et al. (30) reported that the BF% cut-off values were 29.1% for males and 37.2% for females in Caucasians, and 28.3 and 37.1% for male and female African Americans aged 20 years and older using the 1988–1994 NHANES data. The cut-off points from our study were lower than those from aforementioned European and American studies. The ethnicity, to some extents, explains the disparity of body fat between Chinese and White populations, but previous studies also indicated that Asians had relatively higher body fat percentage which predisposed them to prediabetes and diabetes at the given BMI compared to other ethnic groups (16, 31).

Further, the BF% cut-off points in the present study were slightly different from those in other Asian populations. For example, among 10, 774 middle-aged Japanese males (mean age: 47.4 ± 5.7 years), the BF% value for detecting participants with diabetes risk was estimated to be 23.2% (15). In a study of 41, 088 Korean adults aged 18–92 years, the optimal cut-off points were 21.0% for males and 37.0% for females to predict the risk of obesity-related cardiovascular disease (13). In the Chinese population, Jia et al. (32) used the 2007–2008 CNDMS data of 23,769 participants aged 20 or older to evaluate the optimal BF% cut-off values, the results showed that the value was 24.5% in males and 35.7% in females with the diabetes being the endpoints. Among 3,961 subjects aged 30–70 years of Shanghai Diabetes Studies, the cut-off points for the detecting people with risk of T2D were 25.0 and 35.0% for males and females, respectively (33). The optimal total BF% cut-off points in our study were lower in males and higher in females than those reported by the existing studies, as well as others (34, 35). These variations might result from disparity in ages, socio-economic status, dietary culture and eating behavior, lifestyle factors of study population. Also they may be due to methodologies controlling for potential confounders for the ROC curves in our study and different methods to determine the optimal cut-off values, and the outcomes across the studies.

In present study, total BF% level above 21.1% (quintile 3) was significantly associated with the increased risk of T2D in males, while females only showed the significant association between the fifth quintile (38.6%) and the risk of T2D. The results were consistent with a Korean study (36), and suggested sex difference in the influence of BF% on the pathogenesis of T2D. The hormonal difference between males and females greatly impacts body fat distribution, with more lean mass in males and higher fat mass in females for a given BMI (37). It was also reported that the fat was mainly distributed in the trunk for males, while the fat tended to be deposited in the limbs and hips for females, especially in the lower body (38). These above-mentioned findings in body composition, in conjunction with the diversities in culture, lifestyle, environment, socioeconomic status, and energy metabolism may be accounted for differences between males and females in risk of T2D (39).

In 2015–2017, the estimated prevalence of diabetes was 11.2% (95% CI: 10.5–11.9%) among adults in mainland China, which was higher among adults aged 50 and older and among males (40). Previous Chinese cohort studies found that subjects with excess BF% were more likely to have an increased risk of developing diabetes, regardless of the BMI status, and suggested that maintaining normal body fat was meaningful to diabetes prevention (10, 41). However, elevated BF% is not included in conditions recommended for screening T2D in the Chinese guideline (20). Of note, a cross-sectional study conducted by Ruan et al. with 85 T2D patients in China, indicated that reducing body fat was an important adjuvant therapy to improve glycemic control among T2D patients with high body fat (42). Therefore, cost-effective actions to maintain appropriate body fat levels and preventing diabetes, such as specific nutrition education, clinical application of BF% based on sex, and lifestyle intervention included targeting diet and physical activity, are meaningful to take to reduce the disease burden related to T2D in the Chinses healthcare setting.

The major strengths of this study include the use of the CHNS 2015–2018 with a large national population-based study sample, longitudinal assessment of risk ratios for T2D according to quintiles of BF%, and to determine the optimal cut-off values of total and trunk BF% for males and females, respectively. Our study directly shows the etiological role of exposure due to the large-scale prospective cohort study, and provides a more precise effect estimate given many advantages of multilevel mixed-effect modeling instead of traditional regression analyses (43). Moreover, we regarded socio-demographic, lifestyle and dietary variables as confounding factors to justify potential bias. However, several limitations of our study should be considered. First, strict inclusion criteria for the study subjects might reduce the representativeness and generalizability of these findings. Second, BF% measured by BIA is affected by individual and environmental factors such as age, BMI, time of measurement, and physical activity (44, 45), and BIA prediction equations vary by population and device used (15). Although our study used the same type of body composition analyzer validated for Chinese and measured BF% under controlled conditions, the current findings might not apply to other populations.

## Conclusion

In conclusion, the present study showed that the risk of T2D significantly increased over specific level of total and trunk BF% in both Chinese males (≥ 21.1 and ≥ 25.5%, respectively) and females (≥ 38.6 and ≥ 34.4%, respectively). The optimal cut-off values of total and trunk BF% for prediction of T2D risk were determined to be 21.9 and 25.2% for males, and 36.7 and 30.3% for females, respectively. These findings are valuable for suitable modification of body fat percentage based on T2D for Chinese population due to the prospective nature and contribute to the development and implementation of public health actions to further improve the disease burden related to T2D in the Chinese healthcare system.

## Data availability statement

The original contributions presented in the study are included in the article/Supplementary material, further inquiries can be directed to the corresponding authors.

## Ethics statement

The studies involving human participants were reviewed and approved by National Institute for Nutrition and Health, Chinese Center for Disease Control and Prevention. The patients/participants provided their written informed consent to participate in this study.

## Author contributions

Conceptualization and methodology: SZ, ZW, and HJ. Formal analysis and writing-original draft preparation: SZ. Writing-review and editing: ZW, XJ, and JZ. Investigation: HJ, LW, XJ, and JZ. Statistical expertise: HW and BZ. Project administration: BZ and GQ. All authors read and approved the final manuscript.

## Funding

This research was supported by grants from National Key Research and Development Plan of China (No. 2020YFC2006300), the National Institutes of Health (NIH) (R01-HD30880, DK056350, R24 HD050924, and R01-HD38700), the NIH Fogarty International Center (5D43TW007709 and 5D43TW009077), Carolina Population Center (5R24 HD050924), University of North Carolina at Chapel Hill, and the National Financial Projects of Public Health Emergency Project-Nutrition Health and Healthy Diet Campaign (No. 131031107000210002).

## Conflict of interest

The authors declare that the research was conducted in the absence of any commercial or financial relationships that could be construed as a potential conflict of interest.

## Publisher's note

All claims expressed in this article are solely those of the authors and do not necessarily represent those of their affiliated organizations, or those of the publisher, the editors and the reviewers. Any product that may be evaluated in this article, or claim that may be made by its manufacturer, is not guaranteed or endorsed by the publisher.
