# Supplementary material for: Longitudinal relationship between body fat percentage and risk of type 2 diabetes in Chinese adults: Evidence from the China Health and Nutrition Survey
Source: Front Public Health. 2022 Nov 29;10:1032130. doi: 10.3389/fpubh.2022.1032130 (PMC9744757; doi:10.3389/fpubh.2022.1032130)
Supplement: Supplementary file 1 [file Table_1.DOCX]

| Supplementary Table 1 Results of the statistical interaction tests between BF% and sex^1^ | | | |
| --- | --- | --- | --- |
|  | DF | Wald *χ^2^* | *P* |
| Sex*total BF%_5 | 4 | 17.91 | 0.001 |
| Sex*trunk BF%_5 | 4 | 26.91 | <0.001 |
| ^1^ DF: degree of freedom, total BF%_5: quintiles of total BF%, trunk BF%_5: quintiles of trunk BF% | | | |
